# High take off left main coronary artery accompanied by multicryptic left ventricle myocardium detected by cardiac computerized tomography in a young male: case report

**DOI:** 10.1186/s12872-021-02341-7

**Published:** 2021-11-20

**Authors:** Özge Ozden Tok, Ignatios Ikonomidis, Konstantinos Papadopoulos, Ömer Göktekin, Gülsüm Bingöl, Giovanni Di Salvo

**Affiliations:** 1 Cardiology Department, Memorial Bahcelievler Hospital, Istanbul, Turkey; 2 Echocardiography Laboratory, 2nd Cardiology Department, Medical School, National and Kapodistrian University of Athens, Attikon University Hospital, Athens, Greece; 3 Echocardiography Laboratory, European Interbalkan Medical Center, Asklypiou 10 str, 57001 Pylaia, Thessaloniki Greece; 4 Department of Pediatric Cardiology, University of Padova, Padua, Italy

**Keywords:** Left ventricular crypts, High take-off coronary arteries, Cardiac CT

## Abstract

**Background:**

Myocardial crypts are discrete, narrow, blood filled invaginations within the left ventricular myocardium and high-take-off coronary artery are rare manifestations where coronary arteries originate above the sinotubuler junction.

**Case presentation:**

A 41-year-old man with multiple coronary artery disease risk factors admitted to our outpatient department with progressive dyspnea and atypical chest pain. Physical examination revealed no pathological findings. His blood examination revealed only mild to moderately high IgE and LDL levels. Transthoracic echocardiography (TTE) was normal. His treadmill test was normal, yet in the 3rd stage of the test he had an atypically located chest pain which was relieved in the resting period. As he had multiple cardiovascular risk factors, we performed a coronary CT angiography to exclude coronary artery disease. Coronary CT angiography(CCTA) demonstrated multiple myocardial crypts, a muscular VSD like defect which were not detectable with TTE and a high take off left main coronary artery (LMCA). After CCTA, we repeated the TTE to investigate the crypts and VSD-like defect which were clear on CCTA, yet a precise TTE hardly showed crypts and didn’t confirm a shunt between the left and right ventricle. We defined the defect as ‘spontaneously closed muscular VSD’. None of these pathologies were clinically relevant with the patient’s symptoms, thus pneumonology started a montelukast therapy for 1 year and we decided to follow up the patient, as multiple crypts may indicate an early phase hypertrophic cardiomyopathy.

**Conclusions:**

Considering that a high take-off LMCA is a congenital anomaly, encountering multiple crypts which are also congenital pathologies, is plausible, as congenital anomalies may accompany eachother. Echocardiography is a very useful, practical imaging tool but regrettably may be suboptimal due to various patient and method related reasons. Target combination of different cardiovascular imaging tools like echocardiography, cardiac CT(CCT), may be utilized in order to ensure a comprehensive diagnosis particularly.

## Background

Myocardial crypts are discrete, narrow, blood-filled invaginations within the left ventricle myocardium [1, 2]. High take-off coronary arteries are rare anomalies and they are in the anomalous origin group of coronary artery anomalies which originate above sinotubular junction as in our case [3]. To the best of our knowledge, in this article, we describe the first case in the literature in which these two pathologies coexist. Crypts, small VSD’s and coronary abnormalities may be difficult to visualize by TTE particularly if the image quality is not good enough as in that of our patient. Our case highlights the importance of a multimodality approach where the diagnostic utility of a target combination of different imaging modalities is greater than the sum of individual tests.

## Case presentation

A 41-year-old man with coronary artery disease risk factors such as smoking, dyslipidemia and family history(his father had a coronary by-pass surgery at the age of 50) admitted to our outpatient department with progressive dyspnea and atypical chest pain in the last 6 months. On physical examination there were no abnormal findings. His blood pressure was 120/80 mmHg and his heart rate was 64 bpm and regular. There was no cardiac murmur. His blood examination was completely normal except fort he mild to moderately high IgE(400 KU/L) and LDL levels (165 mg/dl).

TTE demonstrated normal systolic function with a left ventricle ejection fraction of 65%, normal sized heart chambers and no valvular or pericardial pathologies. His treadmill test was electrocardiographically normal on exercise and during the resting period, his blood pressure went up to 167/88 mmHg and decreased gradually. He reached 86% of his target heart rate at the 3th stage and succeded 10.5 METS. However at the end of 3rd stage of the test he had an atypical, linear, sharp, left sided chest pain which completely released during the resting period in 2–3 min.

**Fig. 1:**
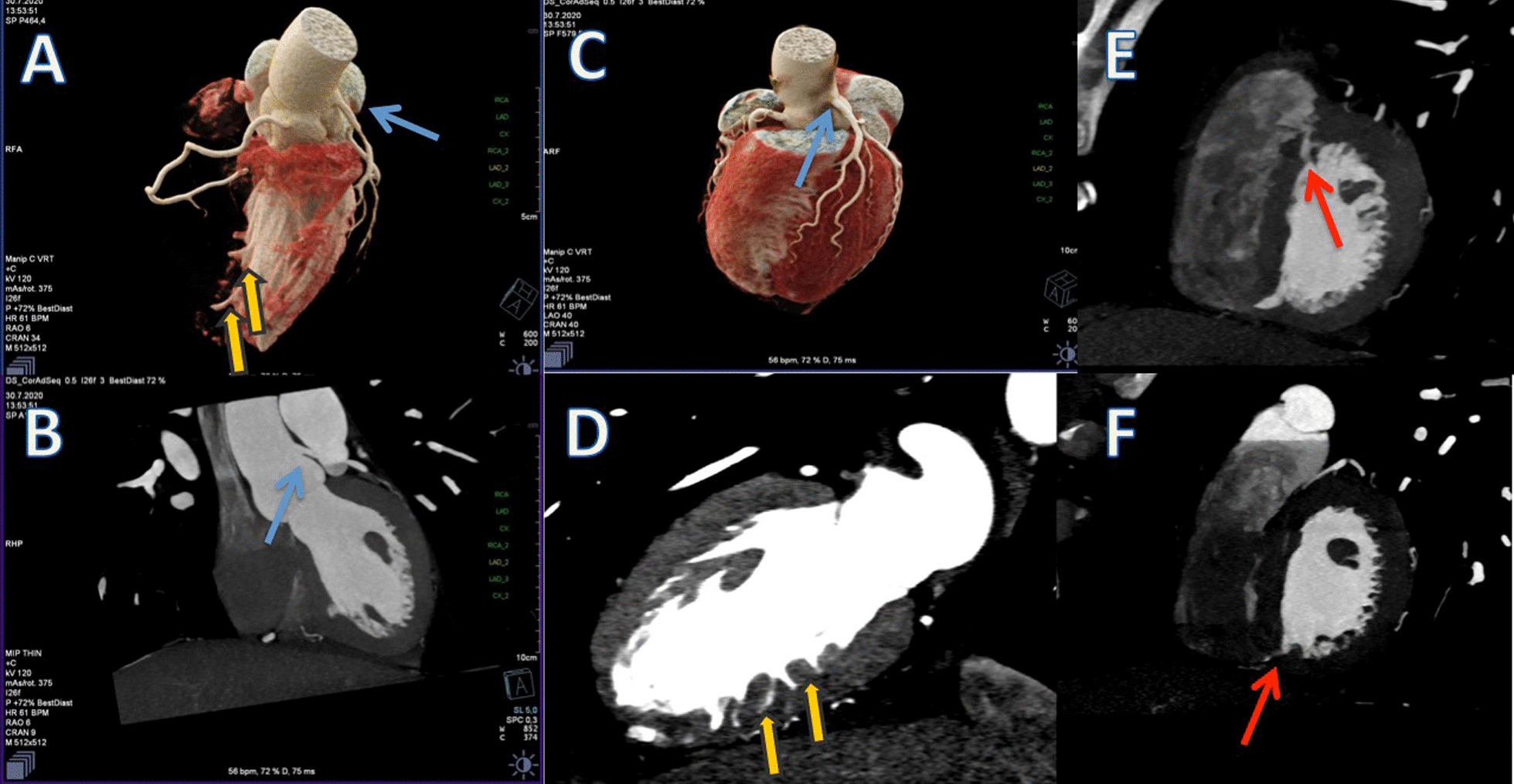
**A** Cardiac CT 3D volume rendering image demonstrates the high take-off LMCA(blue arrow) and multiple crypts in the interventricular septum(yellow arrows). **B** Cardiac CT demonstrates high take-off LMCA(blue arrow). **C** Cardiac CT 3D volume rendering image demonstrates the high take-off LMCA from another view. **D** Cardiac CT 2 chamber view demonstrates the multiple crypts in the inferior wall of the left ventricle(yellow arrows). **E** Cardiac CT short axis view shows the deep crypt in the anterior left–right ventricle insertion point(red arrow). **F** Cardiac CT short axis view shows the deep crypt in the inferior left–right ventricle insertion point(red arrow)

**Fig. 2:**
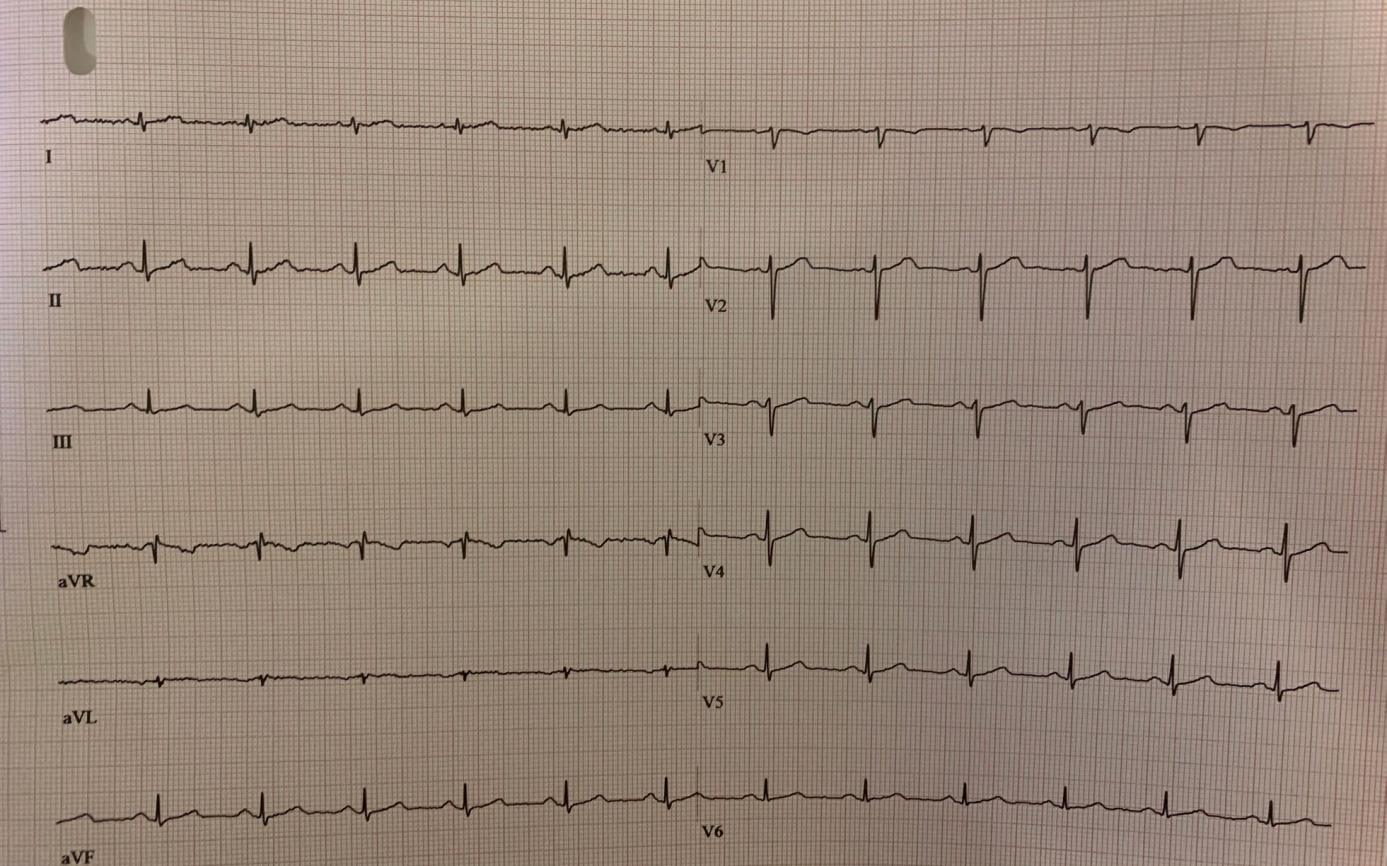
ECG of the patient

As he had strong cardiovascular risk factors and chest pain on exercise, we suggested a CCTA to exclude coronary artery disease. CCTA showed no coronary artery disease, but it revealed a high take off LMCA(originating 2 cm above the aortic annulus and 0.4 mm above the sinotubuler junction) (Fig. 1A, B, C-blue arrow), a rare coronary anomaly, 4 additional myocardial crypts (Fig. 1A, D-yellow arrows), and a muscular VSD-like defect (1E, F-red arrow). All myocardial crypts were within the interventricular septum at different levels. After careful and precise TTE assessment we were convinced that VSD-like defect was a spontaneously closed VSD with a 2 mm width, with no shunt from the left ventricle to right ventricle with CW doppler despite a very deep indentation penetrating the adjoint myocardium. Lack of a cardiac murmur supported our final diagnosis as well. However, all of these were coincidental findings that were detected with CCTA and none of them were clinically relevant with the patient’s symptoms. After a spirometry test, pneumonology associated his dyspnea with his allergic status and started a montelukast therapy for 1 year. Because of normal wall thickness, normal ECG (Fig. 2) and no family history of sudden cardiac death we didn’t consider the ‘early phase hypertrophic cardiomyopathy’ to be first in line for the differnatial diagnosis and didn’t perform a CMR at that point; because it’s pretest probablity was not high but we decided to follow up him yearly and perform a CMR when needed.

High take-off LMCA was considered to be a benign anomaly in this case, as it was not originating from a very high point above the sinuses of Valsalva but just above it and exercise test showed neither an ischemic ECG finding and arrhythmia nor a typical ischemic symptom to support a malignant nature. After all clinical and imaging assessments, we determined that high take-off of LMCA was clinically benign in this particular case and didn’t recommend any spesific precautions. We also didn’t suggest starting a statin therapy and gave him a healthy diet and exercise program to decrease his LDL level.

## Discussion and conclusions

Myocardial fiber or fascicle disarray may result in myocardial crypts, which are congenital abnormalities that have been described in both healthy volunteers and hypertrophic cardiomyopathy patients. [2] Crypts are described as architectural abnormalities of the left ventricle which are discrete and ‘V’ or ‘U’ shaped, penetrating half thickness of the adjacent compact myocardium and which narrows, sometimes even occludes in the systolic phase, without a significant segmental wall motion abnormality [4].

A study demonstrated that crypts may be helpful in detecting gene carriage for hypertrophic cardiomyopathy (HCMP) before the developement of hypertrophy, which facilitates in selecting patients for a close scrutiny [5], because such abnormalities may precede as a “prephenotypic” indicator of HCMP whereas ECG changes, biomarkers for diffuse fibrosis, and advanced echocardiographic techniques are the other established prephenotypic markers in the familial context with a pretest probability of 50% [4].

In the abovementioned study [5] the authors found no crypts in normal individuals, while some other researchers showed sole basal inferior clefts and septal crypts in 6% and 5% of normal subjects using CMR, respectively. Furthermore, they found a higher rate of crypts particularly in congenital heart diseases [6].

In another study, Petryka et al. demonstrated a prevalence of 15.6% in HCM, 13.6% of hypertensive patients and 6% of healthy volunteers [7] suggesting them to be only “innocent bystanders.” It has been also mentioned that spontaneous closure of a ventricular septal defect which we have in our case as well, may resemble a septal cleft [8].

Coronary artery anomalies are also congenital and they are classified into three groups including anomalous origin, anomalous course and anomalous termination. High take-off coronary arteries above sinotubular junction as in our case, are in the anomalous origin group [3]. These are very rare anomalies and found in 5 of 126,000 serial coronary angiographies [9].

The definition of high take-off coronary artery remains debatable. If this definition includes all cases with a coronary ostium located above the sinotubular junction, many cases such as our case with a small distance between the STJ and coronary ostium must be accepted as clinically normal, therefore some researchers have proposed different ‘significant’ high take off distances like 5 mm [10], 1 cm [11], 2 cm [12] above the sinotubular junction.Furthermore, some researchers consider high take-off coronary arteries to be a benign condition [9], while some others believe that it may cause ischemia and sudden cardiac death [10, 12, 13].

To the best of our knowledge our case is the first case in the literature, in which high take-off coronary artery, a spontaneously closed VSD and multiple crypts coexist.

TTE is a very useful and practical tool in our daily practice, but unfortunately sometimes image quality may be suboptimal due to patient related or technical reasons. In the era of cardiovascular multimodality imaging, other imaging tools like CCT, CMRI should be utilized in order to ascertain the diagnosis,particularly when there is a clinical suspicion. Additionally, while evaluating CCTA which is mainly performed for coronary artery disease evaluation, radiologists and cardiologists must double check all other structures comprehensively in order not to overlook a congenital anomaly, myocardial, valvular, pericardial disease and extracardiac findings. In our case, based on CCT findings, we had to perform a target TTE in order to exclude a transventricular shunt. Our case highlights the importance of a multimodality approach where the diagnostic utility of a target combination of different imaging modalities is greater than the sum of individual tests.

## Data Availability

The datasets used and/or analysed during the current study are available from the corresponding author on reasonable request.

## Abbreviations

TTE: Transthoracic echocardiography
CCTA: Coronary Computed Tomography Angiography
VSD: Ventricular Septal Defect
LMCA: Left Main Coronary Artery
ECG: Electrocardiography
CMR: Cardiac Magnetic Resonance
HCMP: Hypertrophic Cardiomyopathy
